# 6-Chloro-3-nitro-*N*-(propan-2-yl)pyridin-2-amine

**DOI:** 10.1107/S1600536811018083

**Published:** 2011-05-20

**Authors:** Xiao-Yu Qing, Yun-Chuang Huang, Ling-Ling Yang, Yong-Mei Xie

**Affiliations:** a State Key Laboratory of Biotherapy and Cancer Center, West China Hospital, West China Medical School, Sichuan University, Chengdu 610041, People’s Republic of China; b Department of Applied Chemistry, College of Chemical Engineering, Sichuan University, Chengdu 610041, People’s Republic of China

## Abstract

There are two mol­ecules in the asymmetric unit mol­ecule of the title compound, C_8_H_10_ClN_3_O_2_. Intra­molecular N—H⋯O hydrogen bonds stabilize the mol­ecular structure. There are no classical inter­molecular hydrogen bonds in the crystal structure.

## Related literature

For the biological activity of 6-chloro-*N*-isopropyl-3-nitro­pyridin-2-amine derivatives, see: Lan *et al.* (2010 ▶); Bavetsias *et al.* (2010 ▶). For bond-length data, see: Allen *et al.* (1987 ▶).
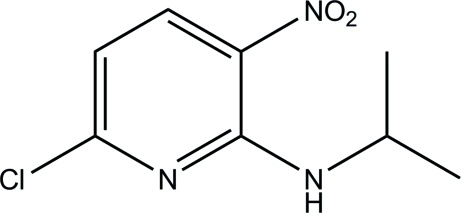


## Experimental

### 

#### Crystal data

C_8_H_10_ClN_3_O_2_
*M* *_r_* = 215.64
Triclinic, 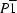

*a* = 7.4283 (8) Å
*b* = 8.9573 (10) Å
*c* = 15.4301 (17) Å
α = 89.672 (9)°
β = 86.252 (9)°
γ = 78.860 (9)°
*V* = 1005.16 (19) Å^3^
*Z* = 4
Mo *K*α radiation
μ = 0.36 mm^−1^
*T* = 295 K
0.28 × 0.23 × 0.18 mm

#### Data collection

Oxford Diffraction Xcalibur Eos diffractometer
Absorption correction: multi-scan (*CrysAlis PRO*; Oxford Diffraction, 2006 ▶) *T*_min_ = 0.971, *T*_max_ = 1.0
8239 measured reflections
4073 independent reflections
2773 reflections with *I* > 2σ(*I*)
*R*_int_ = 0.022

#### Refinement

*R*[*F* ^2^ > 2σ(*F* ^2^)] = 0.061
*wR*(*F* ^2^) = 0.149
*S* = 1.07
4073 reflections
257 parameters
H-atom parameters constrained
Δρ_max_ = 0.24 e Å^−3^
Δρ_min_ = −0.21 e Å^−3^

### 

Data collection: *CrysAlis PRO* (Oxford Diffraction, 2006 ▶); cell refinement: *CrysAlis PRO*; data reduction: *CrysAlis PRO*; program(s) used to solve structure: *SHELXS97* (Sheldrick, 2008 ▶); program(s) used to refine structure: *SHELXL97* (Sheldrick, 2008 ▶); molecular graphics: *OLEX2* (Dolomanov *et al.*, 2009 ▶); software used to prepare material for publication: *OLEX2*.

## Supplementary Material

Crystal structure: contains datablocks I, global. DOI: 10.1107/S1600536811018083/rk2268sup1.cif

Structure factors: contains datablocks I. DOI: 10.1107/S1600536811018083/rk2268Isup2.hkl

Additional supplementary materials:  crystallographic information; 3D view; checkCIF report

## Figures and Tables

**Table 1:** Hydrogen-bond geometry (Å, °)

*D*—H⋯*A*	*D*—H	H⋯*A*	*D*⋯*A*	*D*—H⋯*A*
N2—H2⋯O2	0.86	2.02	2.660 (3)	130
N5—H5⋯O3	0.86	2.01	2.653 (3)	130

**Fig. 1.**
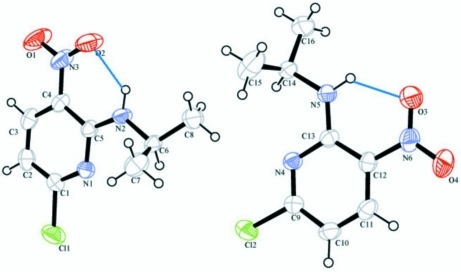
The molecular structure of the title compound with the atom numbering scheme. Displacement ellipsoids are drawn at the 30% probability level. Intramolecular hydrogen bonds are presented by blue lines.

**Fig. 2.**
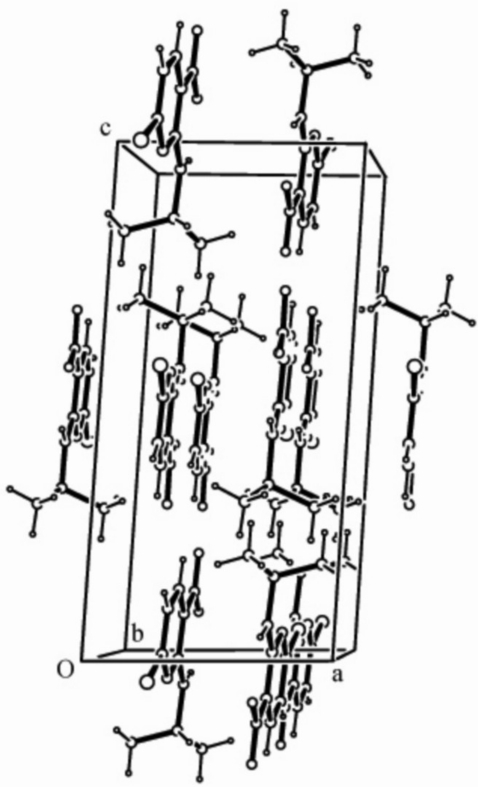
A packing diagram of the title compound.

## supplementary crystallographic information

### Comment

6–Chloro–*N*–isopropyl–3–nitropyridin–2–amine derivatives are of great importance owing to their anticancer activity (Lan *et al.*, 2010; Bavetsias *et al.*, 2010). The title compound is one of the key intermediates in our synthetic investigations of anticancer drugs. Now we synthesized the title compound and report here its molecular and crystal structures. In the title compound, C_8_H_10_ClN_3_O_2_, (Fig. 1), the bond lengths and angles are within normal ranges (Allen *et al.*, 1987). In crystal, there are two molecules of the compound observed in the asymmetric unit. The intramolecular hydrogen bond N2—H2···O2 (N2···O2 = 2.660Å) stabilizes the almost coplanar arrangement of the N2—C5—C4—N3—O2 plane and pyridine ring. And there are no classical hydrogen bonds observed in the crystall packing (Fig. 2).

### Experimental

A solution of 0.58 g (3.0 mmol) of 2,6–dichloro–3–nitropyridine in 20 ml of dichloromethane was stirred in the ice–water bath for a few minutes, then 0.38 ml (4.5 mmol) isopropylamine was added dropwise. The reaction was stirred in the ice–water bath for 4 h, concentrated under reduced pressure and purified by silica gel column chromatography. Crystals suitable for X–ray analysis were obtained by slow evaporation from a solution of dichloromethane.

### Refinement

All H atoms were positioned geometrically and refined as riding (C—H = 0.93Å–0.98Å, N—H = 0.86Å) and allowed to ride on their parent atoms, with *U*_iso_(H) = 1.2–1.5*U*_eq_(parent).

### Crystal data

**Table:**

C_8_H_10_ClN_3_O_2_	*Z* = 4
*M**_r_* = 215.64	*F*(000) = 448
Triclinic, *P*1	*D*_x_ = 1.425 Mg m^−^^3^
Hall symbol: -P 1	Mo *K*α radiation, λ = 0.7107 Å
*a* = 7.4283 (8) Å	Cell parameters from 2782 reflections
*b* = 8.9573 (10) Å	θ = 3.0–29.2°
*c* = 15.4301 (17) Å	µ = 0.36 mm^−^^1^
α = 89.672 (9)°	*T* = 295 K
β = 86.252 (9)°	Block, colourless
γ = 78.860 (9)°	0.28 × 0.23 × 0.18 mm
*V* = 1005.16 (19) Å^3^	

### Data collection

**Table:**

Oxford Diffraction Xcalibur Eos diffractometer	4073 independent reflections
Radiation source: fine-focus sealed tube	2773 reflections with *I* > 2σ(*I*)
graphite	*R*_int_ = 0.022
Detector resolution: 16.0874 pixels mm^-1^	θ_max_ = 26.4°, θ_min_ = 3.0°
ω scans	*h* = −9→8
Absorption correction: multi-scan (*CrysAlis PRO*; Oxford Diffraction, 2006)	*k* = −11→11
*T*_min_ = 0.971, *T*_max_ = 1.0	*l* = −19→19
8239 measured reflections	

### Refinement

**Table:**

Refinement on *F*^2^	Primary atom site location: structure-invariant direct methods
Least-squares matrix: full	Secondary atom site location: difference Fourier map
*R*[*F*^2^ > 2σ(*F*^2^)] = 0.061	Hydrogen site location: inferred from neighbouring sites
*wR*(*F*^2^) = 0.149	H-atom parameters constrained
*S* = 1.07	*w* = 1/[σ^2^(*F*_o_^2^) + (0.0511*P*)^2^ + 0.4285*P*] where *P* = (*F*_o_^2^ + 2*F*_c_^2^)/3
4073 reflections	(Δ/σ)_max_ < 0.001
257 parameters	Δρ_max_ = 0.24 e Å^−^^3^
0 restraints	Δρ_min_ = −0.21 e Å^−^^3^

### Special details

**Table:**

Geometry. All s.u.'s (except the s.u. in the dihedral angle between two l.s. planes) are estimated using the full covariance matrix. The cell s.u.'s are taken into account individually in the estimation of s.u.'s in distances, angles and torsion angles; correlations between s.u.'s in cell parameters are only used when they are defined by crystal symmetry. An approximate (isotropic) treatment of cell s.u.'s is used for estimating s.u.'s involving l.s. planes.
Refinement. Refinement of *F*^2^ against ALL reflections. The weighted *R*–factor *wR* and goodness of fit *S* are based on *F*^2^, conventional *R*–factors *R* are based on *F*, with *F* set to zero for negative *F*^2^. The threshold expression of *F*^2^ > σ(*F*^2^) is used only for calculating *R*–factors(gt) *etc*. and is not relevant to the choice of reflections for refinement. *R*–factors based on *F*^2^ are statistically about twice as large as those based on *F*, and *R*–factors based on ALL data will be even larger.

### Fractional atomic coordinates and isotropic or equivalent isotropic displacement parameters (Å^2^)

**Table:**

	*x*	*y*	*z*	*U*_iso_*/*U*_eq_	
Cl1	0.27337 (14)	−0.24866 (9)	0.55983 (6)	0.0755 (3)	
Cl2	0.22248 (13)	0.27284 (9)	0.95299 (6)	0.0649 (3)	
O1	0.2452 (5)	0.3317 (3)	0.30153 (16)	0.1036 (10)	
O2	0.2403 (4)	0.4488 (3)	0.42175 (16)	0.0911 (9)	
O3	0.3152 (4)	0.9485 (3)	1.08072 (16)	0.0773 (7)	
O4	0.3156 (4)	0.8329 (3)	1.20304 (15)	0.0806 (8)	
N1	0.2670 (3)	0.0404 (3)	0.55475 (14)	0.0438 (6)	
N2	0.2640 (4)	0.2945 (3)	0.56967 (15)	0.0510 (6)	
H2	0.2646	0.3818	0.5464	0.061*	
N3	0.2445 (4)	0.3306 (4)	0.38070 (18)	0.0692 (8)	
N4	0.2595 (3)	0.5533 (2)	0.95342 (14)	0.0420 (5)	
N5	0.2928 (4)	0.8005 (3)	0.93459 (15)	0.0504 (6)	
H5	0.3087	0.8849	0.9562	0.060*	
N6	0.3082 (4)	0.8333 (3)	1.12380 (17)	0.0573 (7)	
C1	0.2645 (4)	−0.0767 (3)	0.5049 (2)	0.0476 (7)	
C2	0.2568 (4)	−0.0768 (4)	0.4162 (2)	0.0576 (8)	
H2A	0.2560	−0.1652	0.3850	0.069*	
C3	0.2504 (4)	0.0606 (4)	0.3764 (2)	0.0563 (8)	
H3	0.2456	0.0676	0.3164	0.068*	
C4	0.2512 (4)	0.1893 (3)	0.42550 (18)	0.0487 (7)	
C5	0.2595 (4)	0.1783 (3)	0.51714 (17)	0.0421 (6)	
C6	0.2678 (4)	0.2846 (3)	0.66435 (17)	0.0452 (7)	
H6	0.3556	0.1926	0.6783	0.054*	
C7	0.0831 (4)	0.2722 (4)	0.7059 (2)	0.0684 (9)	
H7B	−0.0062	0.3597	0.6912	0.103*	
H7C	0.0894	0.2673	0.7679	0.103*	
H7A	0.0479	0.1818	0.6853	0.103*	
C8	0.3374 (6)	0.4205 (4)	0.6968 (2)	0.0769 (11)	
H8A	0.4551	0.4237	0.6680	0.115*	
H8C	0.3493	0.4121	0.7583	0.115*	
H8B	0.2520	0.5120	0.6847	0.115*	
C9	0.2499 (4)	0.4388 (3)	1.00420 (19)	0.0439 (7)	
C10	0.2613 (4)	0.4363 (3)	1.0939 (2)	0.0503 (7)	
H10	0.2556	0.3494	1.1264	0.060*	
C11	0.2814 (4)	0.5690 (4)	1.13095 (19)	0.0493 (7)	
H11	0.2892	0.5746	1.1907	0.059*	
C12	0.2901 (4)	0.6956 (3)	1.08053 (18)	0.0428 (6)	
C13	0.2804 (4)	0.6865 (3)	0.98920 (18)	0.0405 (6)	
C14	0.2811 (4)	0.7920 (3)	0.84014 (18)	0.0481 (7)	
H14	0.3504	0.6925	0.8201	0.058*	
C15	0.0870 (5)	0.8048 (6)	0.8159 (2)	0.0928 (14)	
H15C	0.0166	0.9022	0.8341	0.139*	
H15B	0.0344	0.7258	0.8439	0.139*	
H15A	0.0854	0.7946	0.7540	0.139*	
C16	0.3737 (5)	0.9121 (4)	0.7985 (2)	0.0737 (10)	
H16A	0.3745	0.9037	0.7365	0.111*	
H16B	0.4978	0.8982	0.8156	0.111*	
H16C	0.3078	1.0110	0.8169	0.111*	

### Atomic displacement parameters (Å^2^)

**Table:**

	*U*^11^	*U*^22^	*U*^33^	*U*^12^	*U*^13^	*U*^23^
Cl1	0.1077 (7)	0.0471 (5)	0.0766 (7)	−0.0291 (5)	0.0014 (5)	−0.0021 (4)
Cl2	0.0873 (6)	0.0453 (5)	0.0662 (6)	−0.0228 (4)	−0.0058 (4)	0.0016 (4)
O1	0.177 (3)	0.092 (2)	0.0360 (15)	−0.0075 (19)	−0.0197 (17)	0.0146 (14)
O2	0.164 (3)	0.0536 (15)	0.0507 (16)	−0.0076 (16)	−0.0119 (16)	0.0113 (13)
O3	0.128 (2)	0.0505 (14)	0.0553 (15)	−0.0203 (14)	−0.0114 (14)	−0.0059 (12)
O4	0.118 (2)	0.0906 (19)	0.0381 (14)	−0.0327 (15)	−0.0006 (13)	−0.0173 (13)
N1	0.0527 (14)	0.0420 (13)	0.0369 (13)	−0.0108 (10)	−0.0001 (11)	−0.0027 (11)
N2	0.0850 (18)	0.0355 (12)	0.0317 (13)	−0.0100 (12)	−0.0043 (12)	0.0031 (10)
N3	0.096 (2)	0.0656 (19)	0.0395 (17)	0.0025 (16)	−0.0126 (15)	0.0071 (15)
N4	0.0463 (13)	0.0424 (13)	0.0370 (13)	−0.0089 (10)	−0.0009 (10)	0.0026 (11)
N5	0.0793 (18)	0.0376 (13)	0.0359 (13)	−0.0146 (12)	−0.0054 (12)	−0.0011 (11)
N6	0.0691 (18)	0.0601 (17)	0.0418 (16)	−0.0106 (13)	−0.0013 (13)	−0.0112 (14)
C1	0.0488 (17)	0.0441 (16)	0.0514 (19)	−0.0130 (13)	−0.0023 (13)	−0.0034 (14)
C2	0.061 (2)	0.061 (2)	0.051 (2)	−0.0123 (15)	−0.0064 (15)	−0.0181 (17)
C3	0.059 (2)	0.071 (2)	0.0363 (17)	−0.0060 (16)	−0.0079 (14)	−0.0083 (16)
C4	0.0536 (18)	0.0546 (18)	0.0356 (16)	−0.0038 (14)	−0.0066 (13)	0.0015 (14)
C5	0.0476 (16)	0.0429 (16)	0.0344 (15)	−0.0051 (12)	−0.0022 (12)	−0.0028 (12)
C6	0.0665 (19)	0.0372 (15)	0.0311 (15)	−0.0067 (13)	−0.0063 (13)	−0.0017 (12)
C7	0.069 (2)	0.083 (2)	0.049 (2)	−0.0039 (18)	0.0027 (17)	0.0019 (18)
C8	0.129 (3)	0.054 (2)	0.053 (2)	−0.028 (2)	−0.016 (2)	−0.0034 (17)
C9	0.0434 (16)	0.0430 (16)	0.0451 (17)	−0.0083 (12)	−0.0017 (13)	0.0028 (13)
C10	0.0507 (18)	0.0546 (18)	0.0471 (18)	−0.0143 (14)	−0.0029 (14)	0.0154 (15)
C11	0.0485 (17)	0.067 (2)	0.0323 (16)	−0.0125 (14)	0.0005 (12)	0.0043 (14)
C12	0.0435 (16)	0.0474 (16)	0.0367 (16)	−0.0073 (12)	−0.0008 (12)	−0.0055 (13)
C13	0.0426 (15)	0.0404 (15)	0.0367 (15)	−0.0037 (11)	−0.0007 (12)	0.0014 (12)
C14	0.0665 (19)	0.0433 (16)	0.0363 (16)	−0.0139 (14)	−0.0075 (14)	0.0038 (13)
C15	0.072 (3)	0.149 (4)	0.059 (2)	−0.024 (3)	−0.011 (2)	0.024 (3)
C16	0.110 (3)	0.068 (2)	0.051 (2)	−0.036 (2)	−0.005 (2)	0.0099 (18)

### Geometric parameters (Å, °)

**Table:**

Cl1—C1	1.746 (3)	C6—H6	0.9800
Cl2—C9	1.740 (3)	C6—C7	1.500 (4)
O1—N3	1.221 (3)	C6—C8	1.511 (4)
O2—N3	1.231 (3)	C7—H7B	0.9600
O3—N6	1.232 (3)	C7—H7C	0.9600
O4—N6	1.228 (3)	C7—H7A	0.9600
N1—C1	1.308 (3)	C8—H8A	0.9600
N1—C5	1.355 (3)	C8—H8C	0.9600
N2—H2	0.8600	C8—H8B	0.9600
N2—C5	1.329 (3)	C9—C10	1.392 (4)
N2—C6	1.465 (3)	C10—H10	0.9300
N3—C4	1.432 (4)	C10—C11	1.360 (4)
N4—C9	1.297 (3)	C11—H11	0.9300
N4—C13	1.357 (3)	C11—C12	1.381 (4)
N5—H5	0.8600	C12—C13	1.420 (4)
N5—C13	1.333 (3)	C14—H14	0.9800
N5—C14	1.469 (3)	C14—C15	1.496 (4)
N6—C12	1.438 (4)	C14—C16	1.504 (4)
C1—C2	1.374 (4)	C15—H15C	0.9600
C2—H2A	0.9300	C15—H15B	0.9600
C2—C3	1.365 (4)	C15—H15A	0.9600
C3—H3	0.9300	C16—H16A	0.9600
C3—C4	1.384 (4)	C16—H16B	0.9600
C4—C5	1.421 (4)	C16—H16C	0.9600
			
O1—N3—O2	120.7 (3)	C6—C8—H8C	109.5
O1—N3—C4	119.2 (3)	C6—C8—H8B	109.5
O2—N3—C4	120.1 (3)	C7—C6—H6	108.1
O3—N6—C12	119.4 (2)	C7—C6—C8	112.7 (3)
O4—N6—O3	121.6 (3)	H7B—C7—H7C	109.5
O4—N6—C12	118.9 (3)	H7B—C7—H7A	109.5
N1—C1—Cl1	114.6 (2)	H7C—C7—H7A	109.5
N1—C1—C2	126.9 (3)	C8—C6—H6	108.1
N1—C5—C4	118.8 (2)	H8A—C8—H8C	109.5
N2—C5—N1	116.6 (2)	H8A—C8—H8B	109.5
N2—C5—C4	124.6 (3)	H8C—C8—H8B	109.5
N2—C6—H6	108.1	C9—N4—C13	118.6 (2)
N2—C6—C7	111.4 (2)	C9—C10—H10	122.1
N2—C6—C8	108.3 (2)	C10—C9—Cl2	118.0 (2)
N4—C9—Cl2	115.5 (2)	C10—C11—H11	119.7
N4—C9—C10	126.5 (3)	C10—C11—C12	120.5 (3)
N4—C13—C12	118.9 (2)	C11—C10—C9	115.7 (3)
N5—C13—N4	116.6 (2)	C11—C10—H10	122.1
N5—C13—C12	124.4 (2)	C11—C12—N6	117.8 (3)
N5—C14—H14	107.9	C11—C12—C13	119.7 (3)
N5—C14—C15	111.9 (3)	C12—C11—H11	119.7
N5—C14—C16	108.4 (2)	C13—N5—H5	117.7
C1—N1—C5	118.3 (2)	C13—N5—C14	124.6 (2)
C1—C2—H2A	122.0	C13—C12—N6	122.5 (3)
C2—C1—Cl1	118.4 (2)	C14—N5—H5	117.7
C2—C3—H3	120.0	C14—C15—H15C	109.5
C2—C3—C4	120.0 (3)	C14—C15—H15B	109.5
C3—C2—C1	116.0 (3)	C14—C15—H15A	109.5
C3—C2—H2A	122.0	C14—C16—H16A	109.5
C3—C4—N3	117.8 (3)	C14—C16—H16B	109.5
C3—C4—C5	120.0 (3)	C14—C16—H16C	109.5
C4—C3—H3	120.0	C15—C14—H14	107.9
C5—N2—H2	117.6	C15—C14—C16	112.8 (3)
C5—N2—C6	124.8 (2)	H15C—C15—H15B	109.5
C5—C4—N3	122.2 (3)	H15C—C15—H15A	109.5
C6—N2—H2	117.6	H15B—C15—H15A	109.5
C6—C7—H7B	109.5	C16—C14—H14	107.9
C6—C7—H7C	109.5	H16A—C16—H16B	109.5
C6—C7—H7A	109.5	H16A—C16—H16C	109.5
C6—C8—H8A	109.5	H16B—C16—H16C	109.5

### Hydrogen-bond geometry (Å, °)

**Table:**

*D*—H···*A*	*D*—H	H···*A*	*D*···*A*	*D*—H···*A*
N2—H2···O2	0.86	2.02	2.660 (3)	130
N5—H5···O3	0.86	2.01	2.653 (3)	130
